# Supplementary material for: Elucidating the influence of linker histone variants on chromatosome dynamics and energetics
Source: Nucleic Acids Res. 2020 Mar 4;48(7):3591–604. doi: 10.1093/nar/gkaa121 (PMC7144933; doi:10.1093/nar/gkaa121)
Supplement: gkaa121_Supplemental_Files [file gkaa121_supplemental_files.zip › ChromatosomePaper.zip!/ChromatosomePaper/Graphics/L_DNA_sampling-crop.pdf]

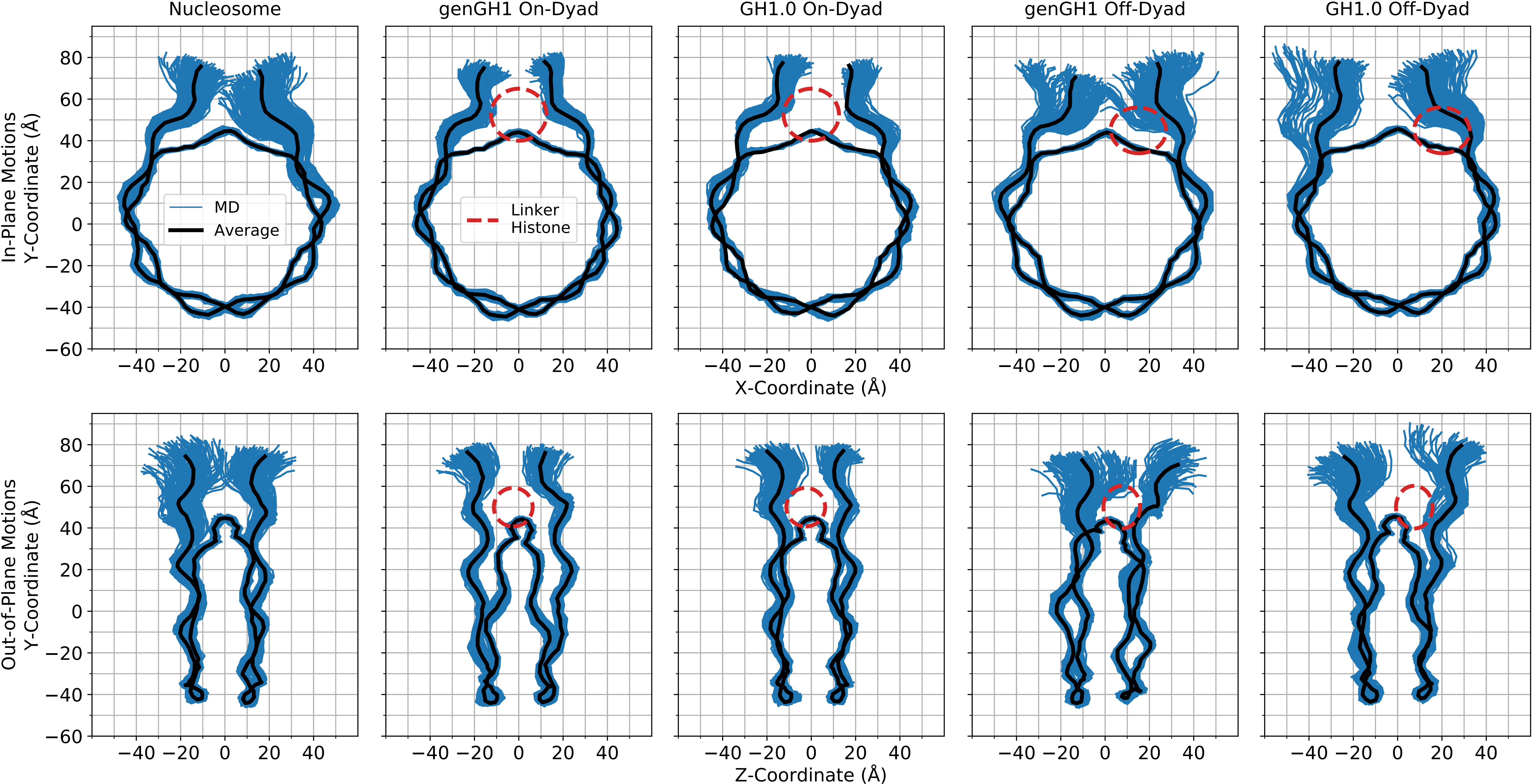

Nucleosome
genGH1 On-Dyad
GH1.0 On-Dyad
genGH1 Off-Dyad
GH1.0 Off-Dyad
In-Plane Motions
Y-Coordinate (Å)
Out-of-Plane Motions
Y-Coordinate (Å)
X-Coordinate (Å)
Z-Coordinate (Å)
MD
Average
Linker
Histone
80
60
40
20
0
−20
−40
−60
−40 −20 0 20 40
