# Supplementary material for: Elucidating the influence of linker histone variants on chromatosome dynamics and energetics
Source: Nucleic Acids Res. 2020 Mar 4;48(7):3591–604. doi: 10.1093/nar/gkaa121 (PMC7144933; doi:10.1093/nar/gkaa121)
Supplement: gkaa121_Supplemental_Files [file gkaa121_supplemental_files.zip › ChromatosomePaper.zip!/ChromatosomePaper/Graphics/alpha_beta-crop.pdf]

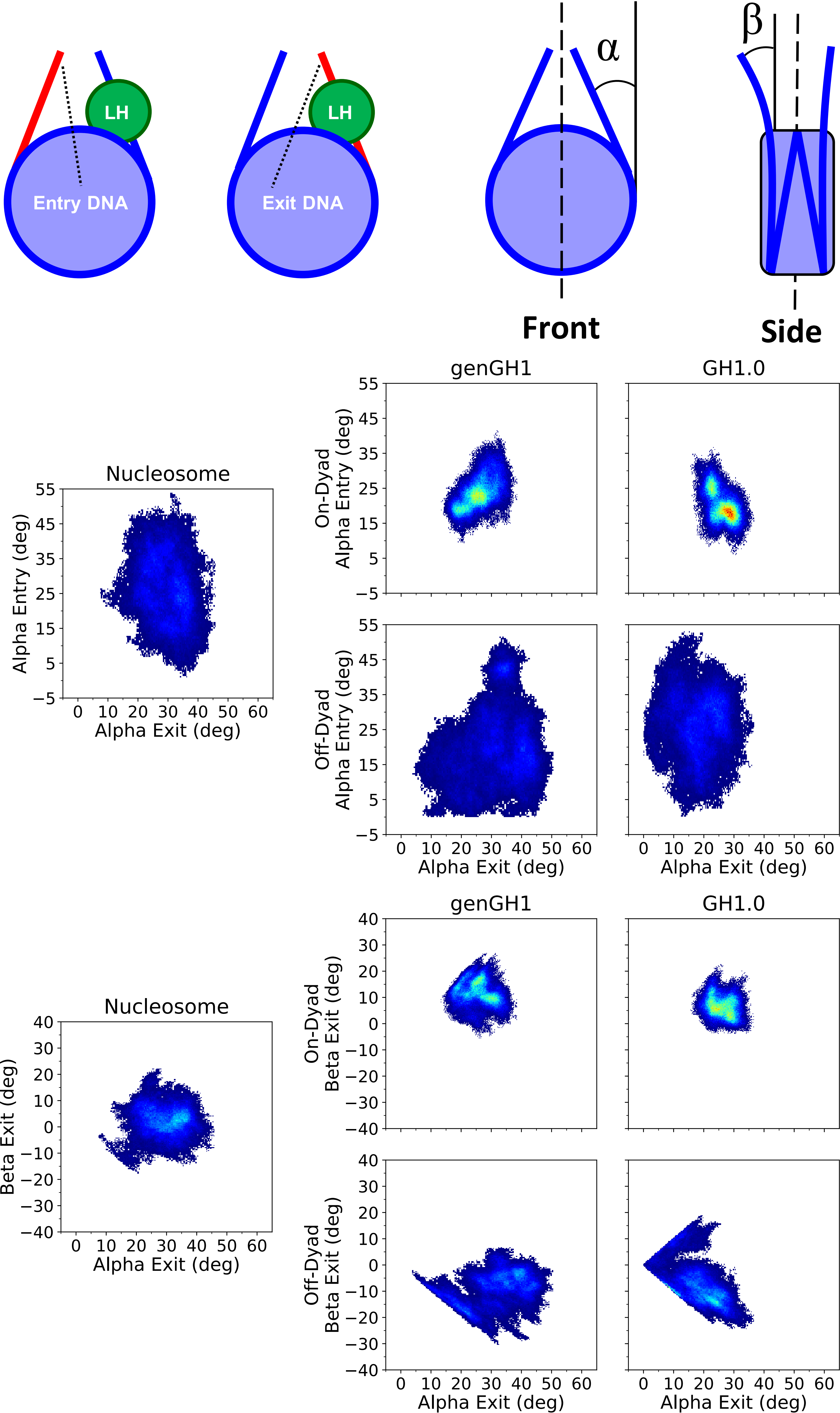

LH
Entry DNA
LH
Exit DNA
α
Front
β
Side
genGH1
GH1.0
Nucleosome
On-Dyad Alpha Entry (deg)
Off-Dyad Alpha Entry (deg)
Alpha Entry (deg)
Alpha Exit (deg)
genGH1
GH1.0
Nucleosome
On-Dyad Beta Exit (deg)
Off-Dyad Beta Exit (deg)
Beta Exit (deg)
Alpha Exit (deg)
