# Supplementary material for: Elucidating the influence of linker histone variants on chromatosome dynamics and energetics
Source: Nucleic Acids Res. 2020 Mar 4;48(7):3591–604. doi: 10.1093/nar/gkaa121 (PMC7144933; doi:10.1093/nar/gkaa121)
Supplement: gkaa121_Supplemental_Files [file gkaa121_supplemental_files.zip › ChromatosomePaper.zip!/ChromatosomePaper/Graphics/chromatosome_pics-crop.pdf]

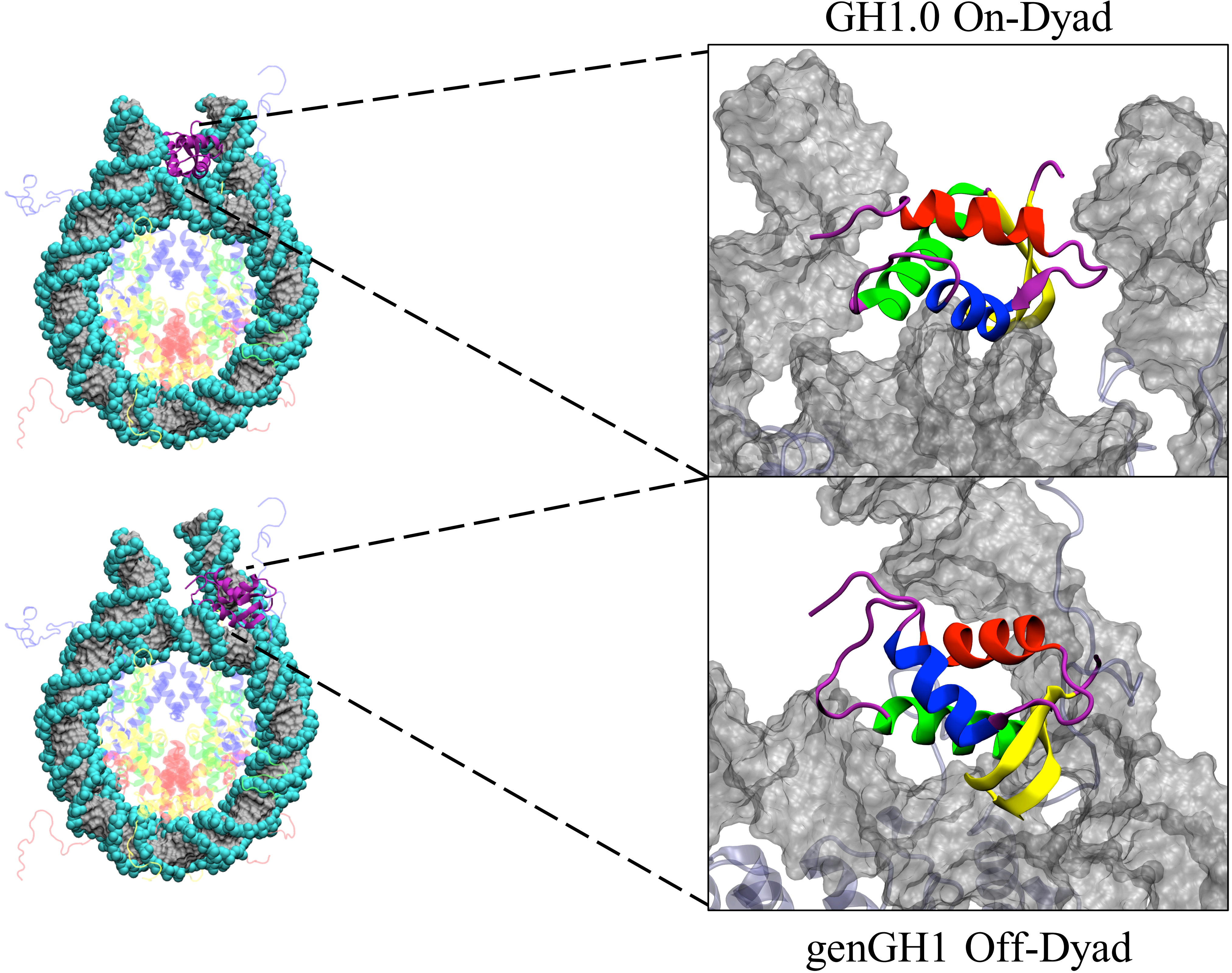


```
genGH1  ⁴³SASHPPTQQ-MIDAAIKNLKERGGSSLLAIKKYITATYKVDAQKLAPFIKKYLKSLVVNGKLIQTKGKGASGSFKLSA¹¹⁹
GH1.0   ²²SASHP-TYSEMIAAAIRAEKSRGGSSRQSIQKYIKSHYKVG-HNADLQIKLSIRRLIAAGVLKQTKGVGASGSFRLAK⁹⁷
```
