# Supplementary material for: Elucidating the influence of linker histone variants on chromatosome dynamics and energetics
Source: Nucleic Acids Res. 2020 Mar 4;48(7):3591–604. doi: 10.1093/nar/gkaa121 (PMC7144933; doi:10.1093/nar/gkaa121)
Supplement: gkaa121_Supplemental_Files [file gkaa121_supplemental_files.zip › ChromatosomePaper.zip!/ChromatosomePaper/Graphics/clusters_compressed.pdf]

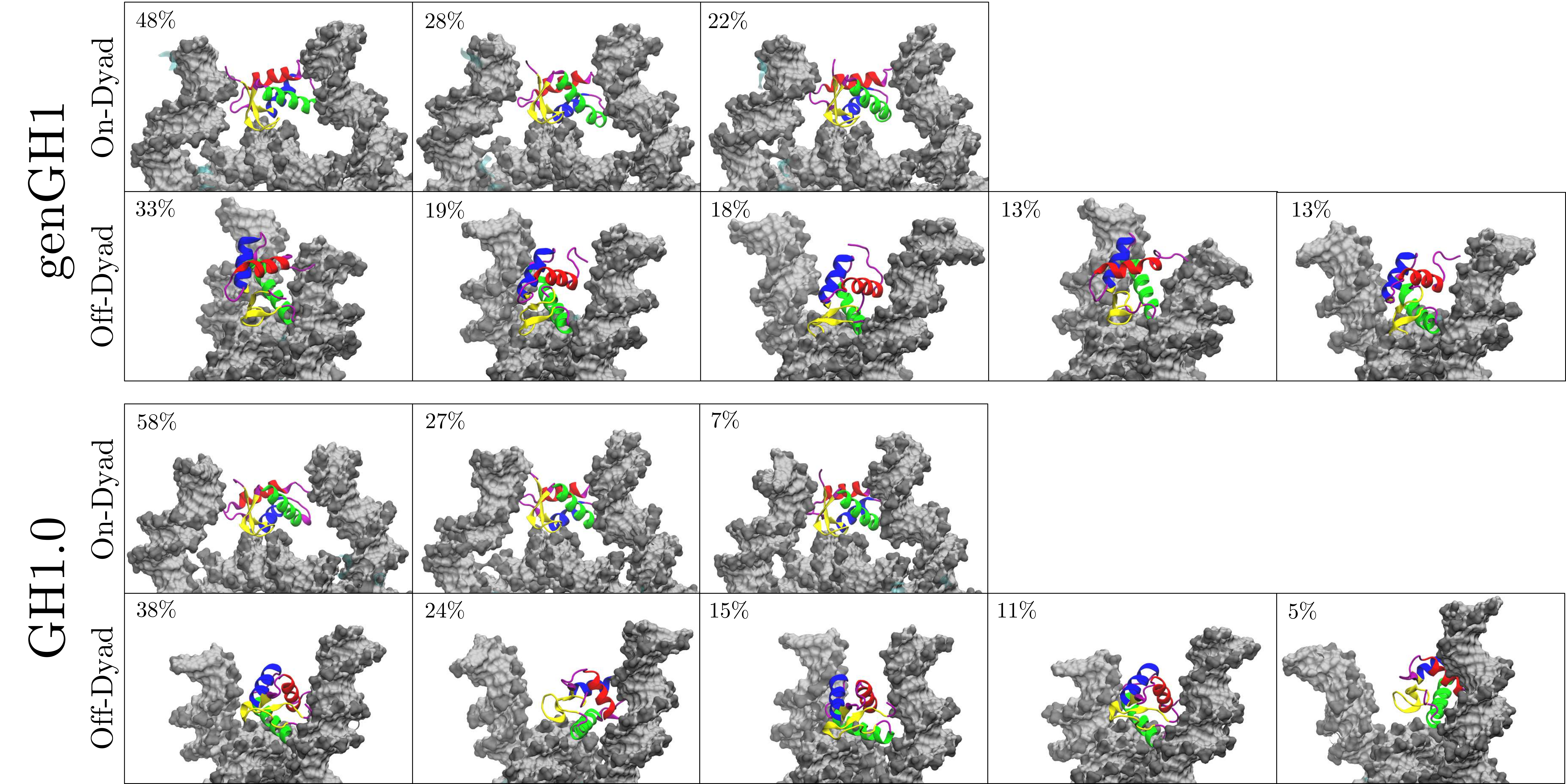
genGH1
On-Dyad
48%
28%
22%
Off-Dyad
33%
19%
18%
13%
13%
GH1.0
On-Dyad
58%
27%
7%
Off-Dyad
38%
24%
15%
11%
5%
