# Supplementary material for: Elucidating the influence of linker histone variants on chromatosome dynamics and energetics
Source: Nucleic Acids Res. 2020 Mar 4;48(7):3591–604. doi: 10.1093/nar/gkaa121 (PMC7144933; doi:10.1093/nar/gkaa121)
Supplement: gkaa121_Supplemental_Files [file gkaa121_supplemental_files.zip › ChromatosomePaper.zip!/ChromatosomePaper/Graphics/PMF_w_PDB_angular_diagram-crop.pdf]

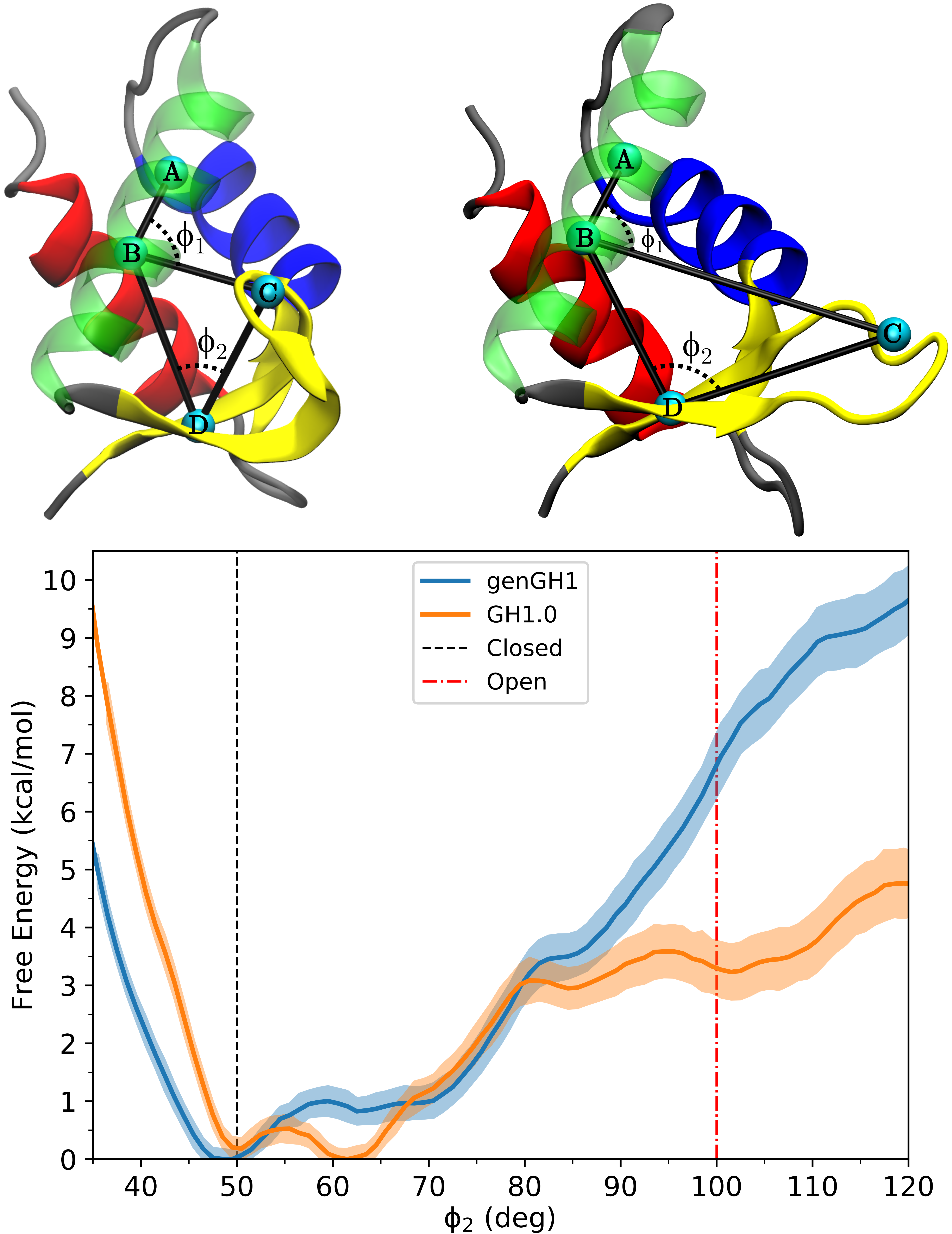
A
B
C
D
ϕ1
ϕ2
A
B
C
D
ϕ1
ϕ2
genGH1
GH1.0
Closed
Open
Free Energy (kcal/mol)
ϕ2 (deg)
0
1
2
3
4
5
6
7
8
9
10
40
50
60
70
80
90
100
110
120
