# Supplementary material for: Elucidating the influence of linker histone variants on chromatosome dynamics and energetics
Source: Nucleic Acids Res. 2020 Mar 4;48(7):3591–604. doi: 10.1093/nar/gkaa121 (PMC7144933; doi:10.1093/nar/gkaa121)
Supplement: gkaa121_Supplemental_Files [file gkaa121_supplemental_files.zip › ChromatosomePaper.zip!/ChromatosomePaper/Graphics/both_h1_h5_contacts.pdf]

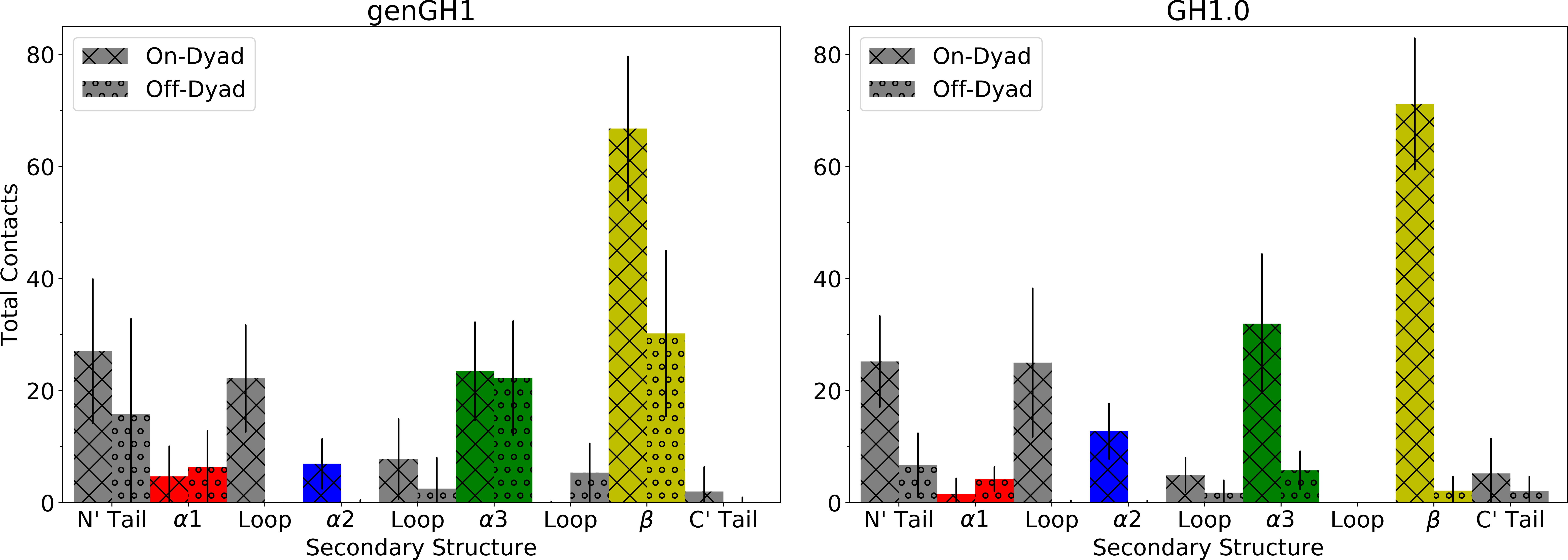
genGH1
GH1.0
On-Dyad
Off-Dyad
Total Contacts
Secondary Structure
N' Tail
α1
Loop
α2
α3
β
C' Tail
0
20
40
60
80
