# Supplementary material for: Elucidating the influence of linker histone variants on chromatosome dynamics and energetics
Source: Nucleic Acids Res. 2020 Mar 4;48(7):3591–604. doi: 10.1093/nar/gkaa121 (PMC7144933; doi:10.1093/nar/gkaa121)
Supplement: gkaa121_Supplemental_Files [file gkaa121_supplemental_files.zip › ChromatosomePaper.zip!/ChromatosomePaper/Graphics/phi1_phi2_plot-crop.pdf]

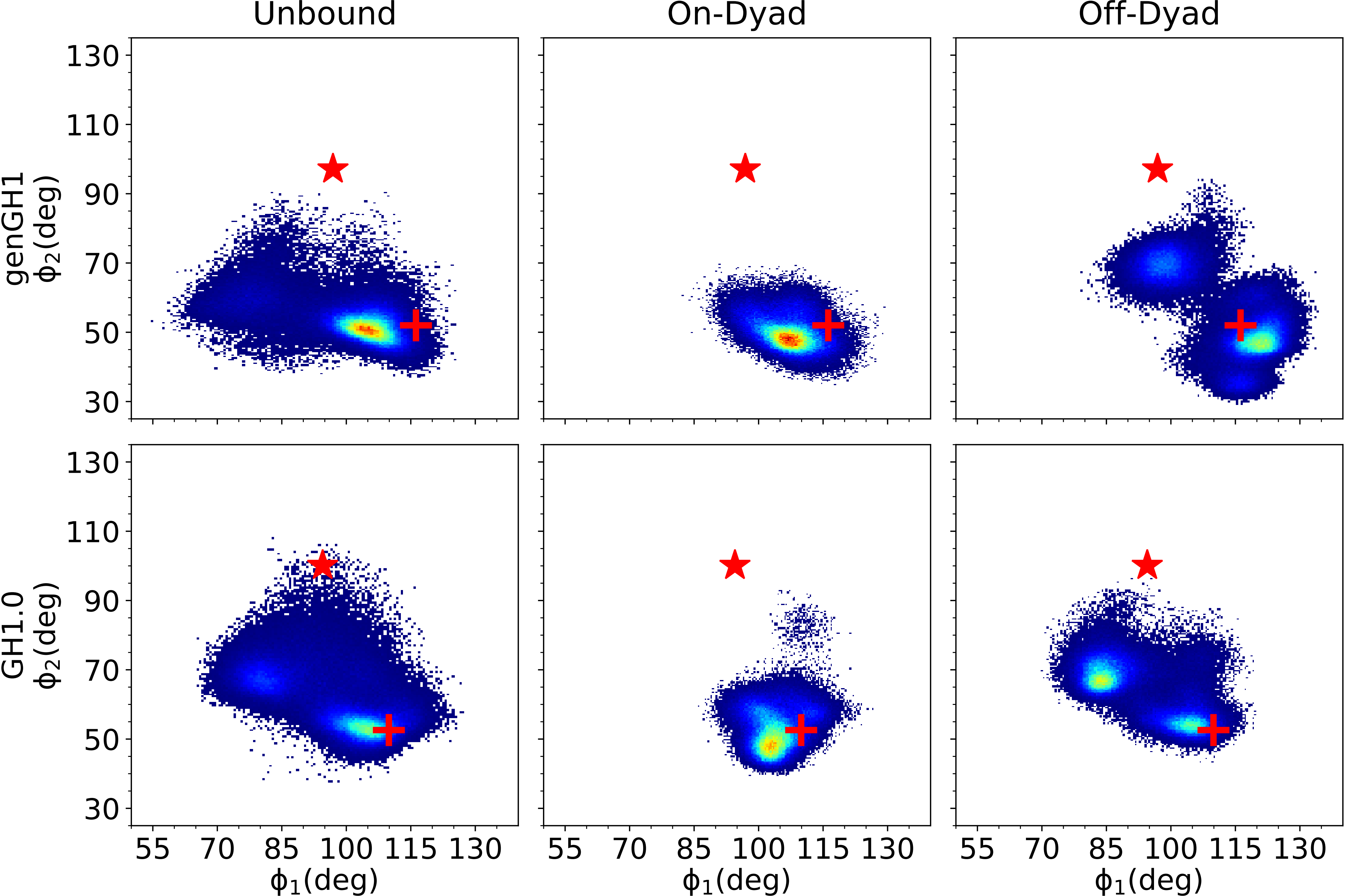
Unbound
On-Dyad
Off-Dyad
genGH1
ϕ_2(deg)
GH1.0
ϕ_2(deg)
130
110
90
70
50
30
55
70
85
100
115
130
ϕ_1(deg)
